# Mediastinal epithelioid sarcoma: a case report

**DOI:** 10.3389/fmed.2025.1701502

**Published:** 2025-10-30

**Authors:** Xianwen Hu, Ronghua Yu, Song Li, Jiong Cai, Dongfeng Pan

**Affiliations:** Department of Nuclear Medicine, Affiliated Hospital of Zunyi Medical University, Zunyi, Guizhou, China

**Keywords:** epithelioid sarcoma, mediastinum, computed tomography, ^18^F-FDG, PET/CT

## Abstract

Epithelioid sarcoma (ES) is a rare soft tissue malignancy of uncertain differentiation that can arise in various anatomical locations; however, its occurrence in the mediastinum is exceptionally uncommon. Herein, we present a 57-year-old woman diagnosed with mediastinal ES. A soft tissue mass in the mediastinum was initially detected incidentally during a routine physical examination; however, the patient did not perceive it as concerning and therefore did not seek further evaluation or treatment. Approximately 5 months later, the patient presented to our hospital with complaints of cough and chest tightness for diagnostic assessment and management. A chest computed tomography (CT) scan revealed a soft tissue density mass in the posterior mediastinum, with visible intratumoral nodular calcifications and mild heterogeneous enhancement on contrast-enhanced CT, findings suggestive of a neurogenic tumor. Subsequently, the patient underwent surgical resection, and postoperative pathological examination confirmed the diagnosis of ES. Unfortunately, during the subsequent imaging follow-up, it was found that the tumor had recurred within a short period of time. ES occurring in the mediastinum is rare, while our case suggests that it should be considered as one of the imaging differential diagnoses for mediastinal or near hilar tumors.

## Introduction

Epithelioid sarcoma (ES) is a mesenchymal malignancy characterized by histological heterogeneity and is classified as a tumor of uncertain differentiation in the 2020 World Health Organization classification of soft tissue and bone tumors (1). The tumor cells exhibit partial or complete epithelioid morphology and immunophenotype, and the disease is often clinically characterized by slowly growing masses in subcutaneous or deep soft tissues (2). Following surgical resection, these tumors are prone to local recurrence or regional metastasis, resulting in a poor prognosis (3). Based on the differences in histological morphology, it can be divided into two subtypes, including classical type ES (CES) and proximal type ES (PES). CES is more common in the dermis or subcutaneous tissue of the distal limb of the adolescent, characterized histologically by multiple granulomatous nodules composed of spindle cells and epithelioid cells, with the majority being epithelioid cells (4). PES is more commonly found in the pelvic, genital, inguinal, gluteal, and deep perineal soft tissues of middle-aged individuals. The tumor consists of large, round epithelioid cells exhibiting varying degrees of striated muscle-like morphology, which are highly invasive and prone to metastasis, leading to a worse prognosis compared to CES (2). Here, we present a case of PES occurring in an unusual location, the mediastinum, focusing on its imaging findings and the aim is to raise awareness of this rare disease.

## Case presentation

**Figure 1:**
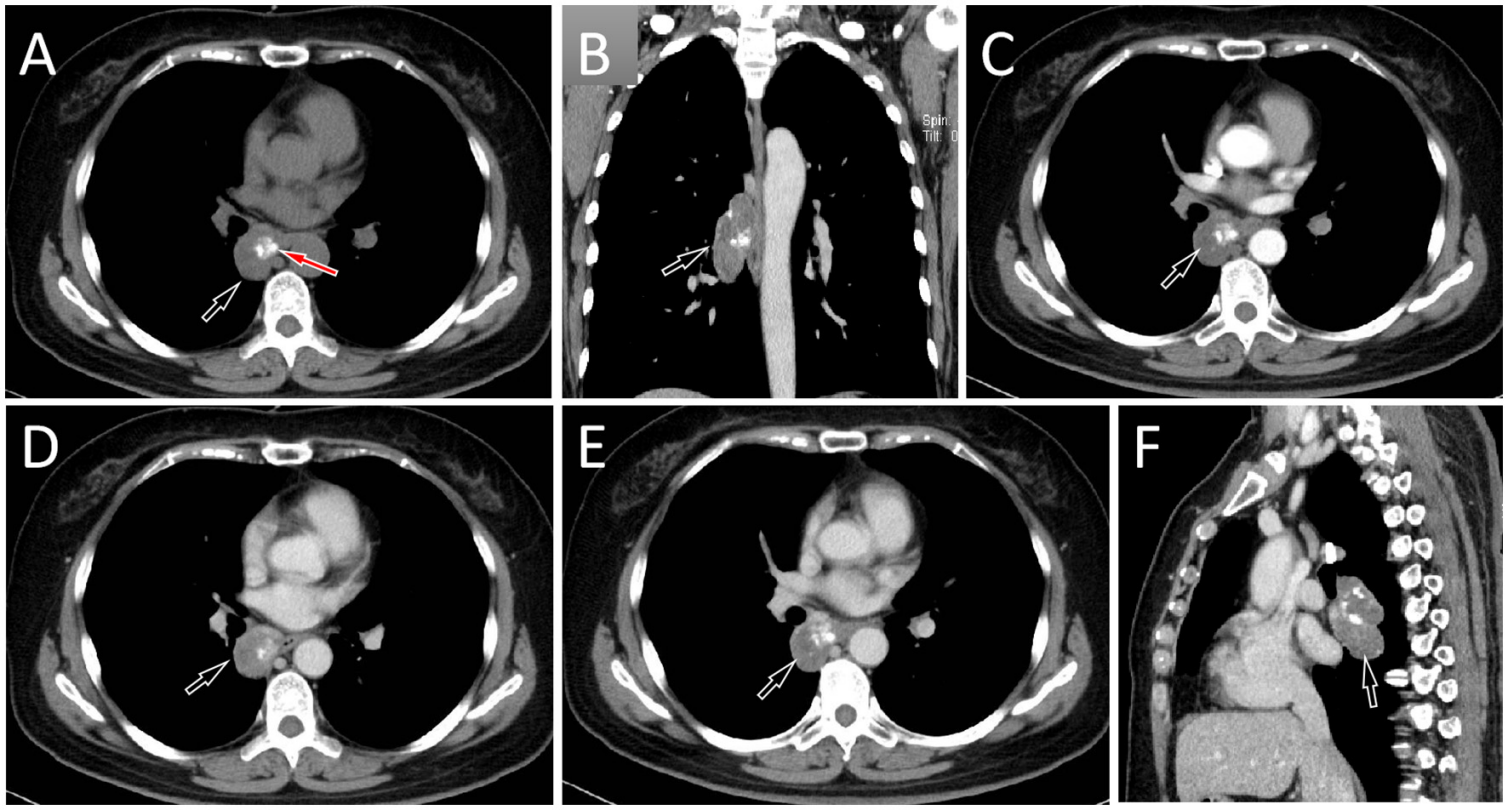
Chest computed tomography (CT) mediastinal window **(A)** revealed a soft tissue density mass (black arrow) about 4.6 cm × 3.2 cm in size with nodular calcification (red arrow) can be observed on the right posterior mediastinum; **(B)** The coronal view shows that the tumor is spindle shaped (arrow); In the arterial phase **(C)** and vein phase **(D)** of contrast-enhanced CT, the lesion showed mild uneven enhancement (arrows); In the delayed phase (**E**, axial, **F**, sagittal), the enhancement of the mass gradually diminishes (arrows).

**Figure 2:**
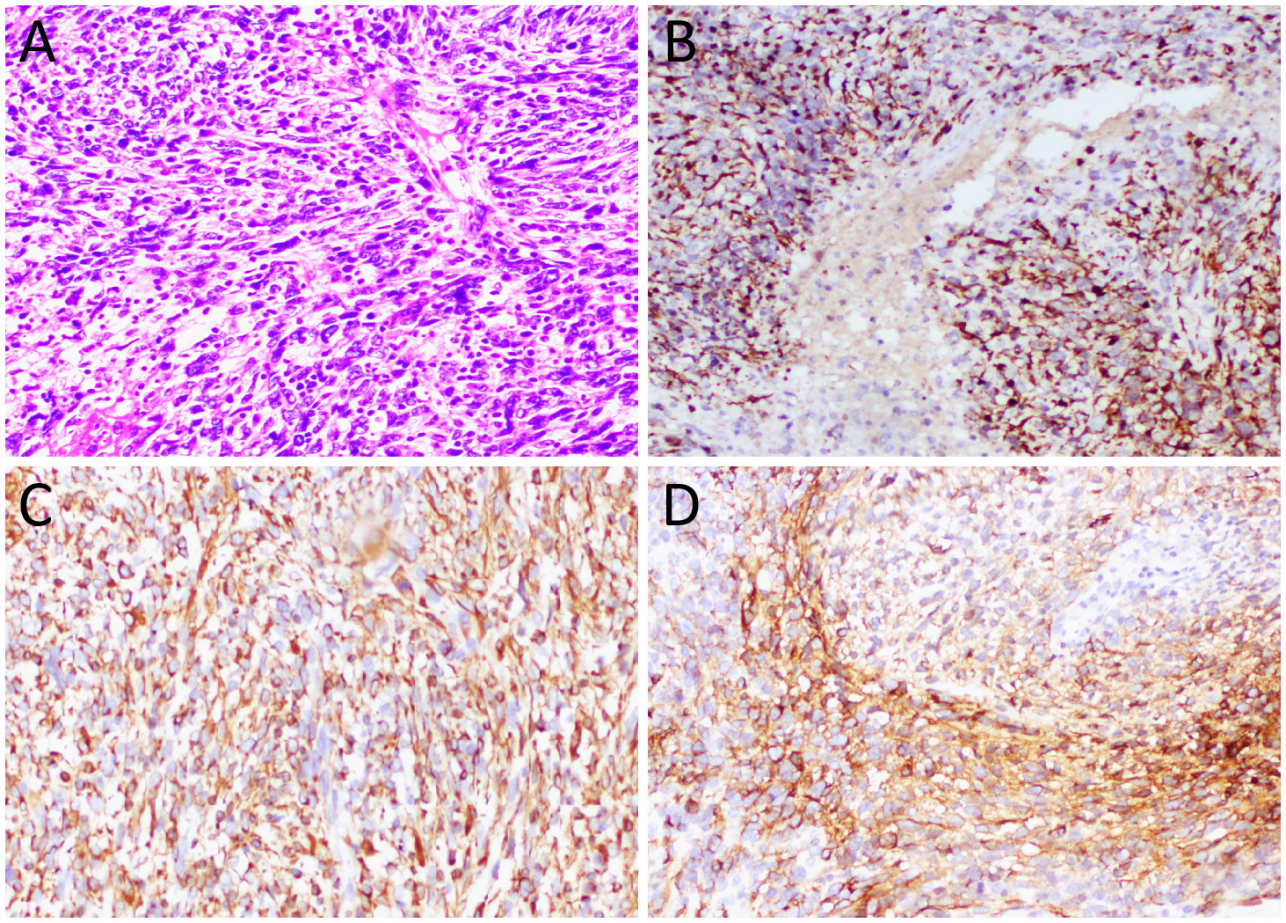
**(A)** Hematoxylin–eosin staining (magnification, 100 × ) showed diffuse nodular epithelioid cells in tumor tissue, accompanied by necrosis and bleeding. Immunohistochemical results revealed that the tumor cells positively expressed CK **(B)**, vimentin **(C)**, and CD34 **(D)**.

**Figure 3:**
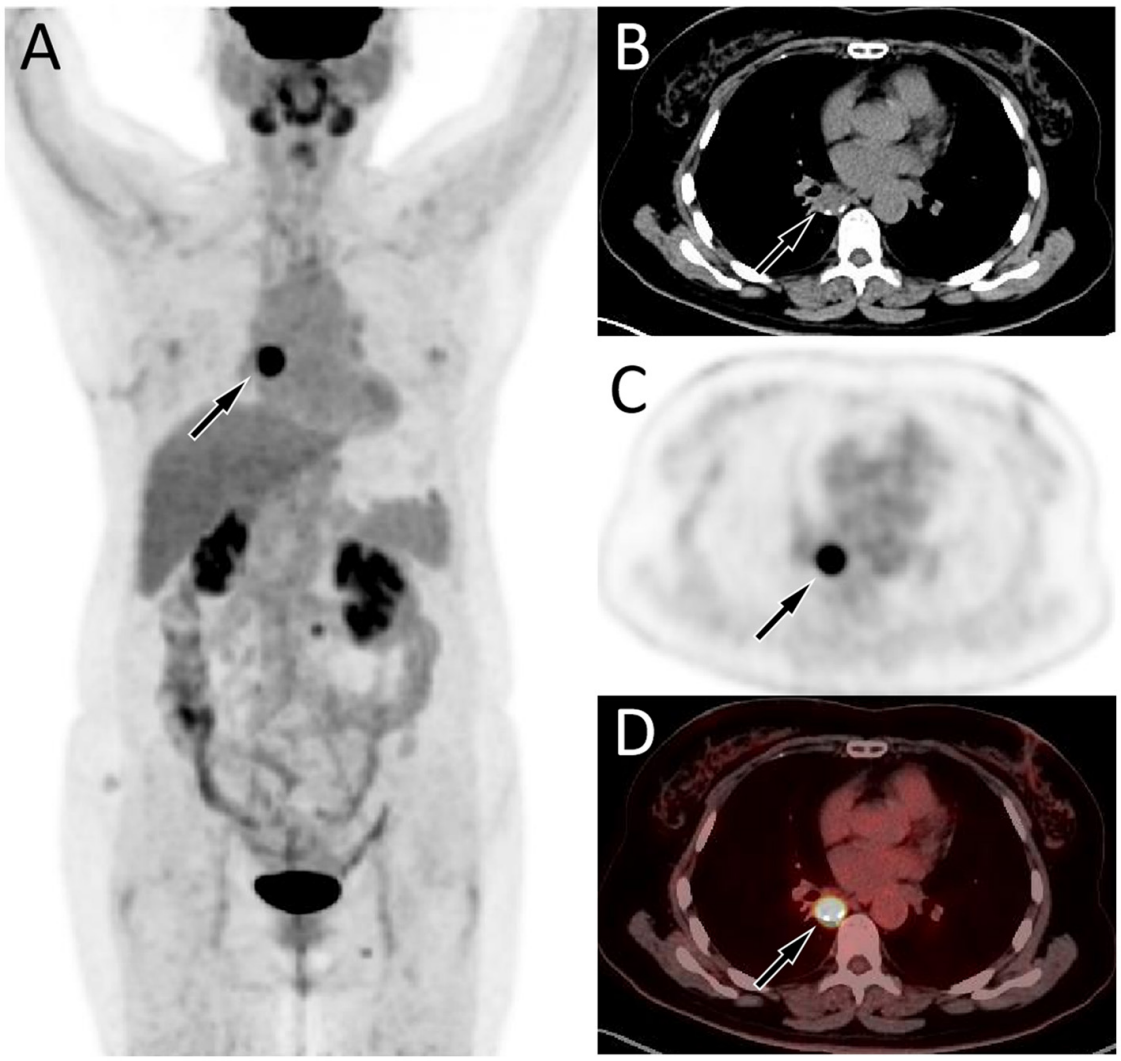
Fluorine-18 fluorodeoxyglucose (^18^F-FDG) positron emission tomography (PET)/CT imaging 4 months after surgery of the patient; the maximum intensity projection (MIP, **A**) showed an obviously increased ^18^F-FDG uptake in the mediastinal region (arrow). Axial CT **(B)** showed a soft tissue mass about 2.1 cm × 1.7 cm in size on the right posterior mediastinum (arrow). The corresponding lesion had obviously increased ^18^F-FDG uptake on axial PET (**C**, arrow) and PET/CT fusion (**D**, arrow), with a maximum standardized uptake value (SUVmax) of 20.3.

**Figure 4:**
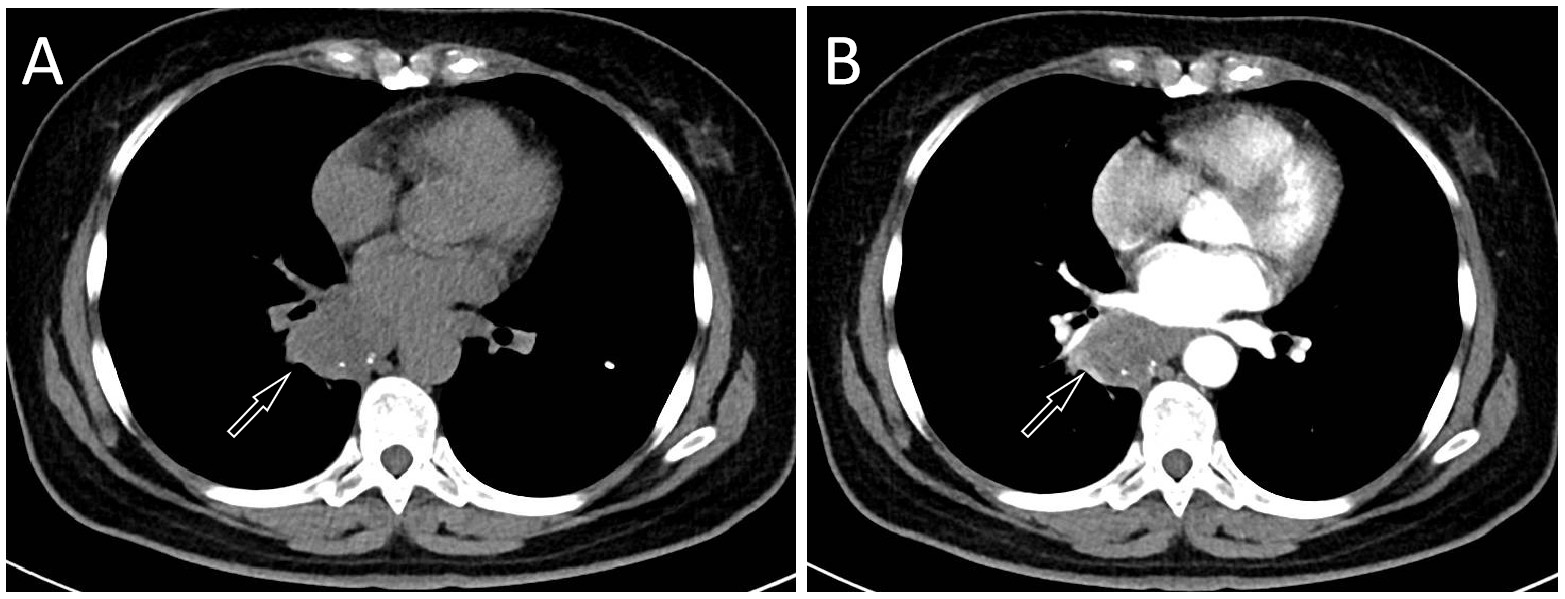
The patient's chest CT 6 months after surgery, CT plain scan **(A)** and contrast-enhanced CT **(B)** revealed a significant increase in the volume of the mass, with a maximum cross-sectional area of about 4.7 cm × 3.9 cm.

A 57-year-old female patient accidentally discovered a soft tissue mass in her posterior mediastinum during a physical examination in an outside hospital on 11 November 2023. She consciously had no obvious clinical symptoms and was not paid attention to, nor was undergoing any treatment. On 20 April 2024, the patient came to our hospital for medical help due to a cough and chest tightness. The physical examination did not reveal any positive signs. She and her family have denied any history of cancer or major genetic disorders. The serological laboratory test results revealed that the treponema pallidum antibody was positive, while other results, including blood routine, liver and kidney function, and serum tumor markers, were all negative. She had a history of syphilis 15 years ago; the condition resolved completely following treatment, and there are currently no clinical or serological indications of recurrence. She denied any history of tuberculosis, hepatitis, or malignancies. The patient underwent chest computed tomography (CT) examination (Figure 1) on April 24, which revealed a well-defined soft-tissue density mass in the posterior mediastinum with calcification. Contrast-enhanced CT showed a slight enhancement of the mass, suggesting a possible neurogenic tumor. The patient underwent surgical complete resection of the tumor through thoracoscopy under general anesthesia on April 26 after completing the preoperative routine examination. During the operation, the tumor was found to be located between the spine, azygos vein, trachea, and esophagus, with a size of approximately 6 cm × 4.5 cm × 4.0 cm, spindle-shaped, with a complete capsule and no obvious adhesion to surrounding structures. The tumor was gradually separated along its edges using an ultrasonic knife until it was completely removed, and then sent for pathological examination. Hematoxylin–eosin staining (as shown in Figure 2) showed that the excised tumor tissue appeared as a gray–red envelope block with intact capsule, medium texture, and calcified lesions in some areas. No tumor invasion was observed at the resection margin. Immunohistochemistry revealed tumor cells positively expressed vimentin, Cluster of Differentiation 34 (CD34), cytokeratin (CK), and CD56, while they negatively expressed insulinoma-associated protein 1 (INSM1), S100, CD68, and so on. Based on these histopathological findings of the patient, she was diagnosed with PES. After surgery, the patient did not receive further radiotherapy, chemotherapy, or other treatment methods. At 4 months after discharge, a chest CT examination revealed a new soft tissue density nodule at the site of her previous surgery. In order to further evaluate the nature of the nodule and determine the next treatment plan, the patient underwent ^18^F-FDG PET/CT imaging (as shown in Figure 3) on August 20. The results showed that the above nodule, as shown on CT, showed a significantly increased ^18^F-fluorodeoxyglucose (^18^F-FDG) uptake, while no significant hot spots were observed in the rest of the body. These imaging findings suggested the possibility of local recurrence of the tumor, and due to the limitation of the lesion, the surgeon planned to perform a second operation on her. However, the patient refused surgery again. On October 16, the patient returned to the hospital for chest pain and underwent chest CT examination (Figure 4), which showed that the mass was significantly larger than before. Currently, she is receiving a chemotherapy regimen of epirubicin in combination with isocyclophosphamide.

## Discussion

ES is a highly aggressive malignant soft tissue tumor; the source of differentiation is unknown, and it accounts for less than 1% of all soft tissue tumors (1). ES can occur in any part of the body, but PES usually occurs in deep organs, mostly located in the pelvis, perineum, genitalia, etc. (5). Unlike CES, PES is more common in adults, and its clinical symptoms are usually related to the mass pressing on the surrounding organs (6, 7). The patient we reported was a 57-year-old female, consistent with the typical age of onset for PES, but the tumor occurred in the mediastinum—a location rarely documented in the literature. The lesion was initially detected incidentally in the absence of clinical symptoms, likely due to the small size of the mass. As the tumor progressed and increased in size, leading to compression or invasion of surrounding tissues, the patient developed symptoms including chest pain and chest discomfort.

Due to the rarity of Ewing sarcoma (ES), imaging studies on this tumor are limited. ES may present as a single or multiple subcutaneous or deep soft tissue masses, exhibiting invasive growth and poorly defined tumor margins. Small lesions appear as round or oval soft tissue density shadows. As the lesion increases in size, it often becomes lobulated and is prone to hemorrhage and necrosis, with occasional calcifications that contribute to heterogeneous density (8, 9). Contrast-enhanced CT of ES can show varying degrees of enhancement, but often presents as significantly uneven enhancement (8). It should be noted that ES occurring in different parts may have different imaging findings, while there is currently no literature published on imaging studies of mediastinal ES. The CT findings in the patient reported in our current study revealed a round, soft tissue mass with nodular areas of high-density calcification. Contrast-enhanced scanning demonstrated mild heterogeneous enhancement, consistent with the typical CT features of Ewing sarcoma (ES). Studies (10, 11) on PET imaging of ES are primarily limited to case reports, all of which have shown significantly increased ^18^F-FDG uptake, suggesting a higher degree of malignancy of the tumor. Our patient underwent a PET/CT examination 4 months after surgery, and the result showed a nodular shadow with increased ^18^F-FDG uptake, indicating tumor recurrence.

Based on the imaging findings of the mediastinal ES reported in this study, differentiation is required from neurogenic tumors in the posterior mediastinum, central lung carcinoid tumors, and central pulmonary sclerosing pneumocytoma (PSP). The CT findings of typical posterior mediastinal neurogenic neoplasms show a single mass near a spine with a regular shape, mostly round or oval, and some may have lobed changes (12). It typically presents as a soft tissue mass with density equal to or slightly lower than that of the chest wall, projecting into one side of the mediastinum, and often contains scattered foci of calcification or necrotic liquefaction within the lesion (13). Benign neurogenic tumors typically exhibit well-defined margins, and contrast-enhanced scans usually demonstrate homogeneous enhancement. In contrast, malignant neurogenic tumors are often larger, display ill-defined borders with invasion into surrounding tissues, and show marked heterogeneous enhancement on contrast-enhanced imaging (14). The current patient's contrast-enhanced CT demonstrates mild heterogeneous enhancement, which differs from the typical imaging pattern. Central lung carcinoid tumors typically present as round or oval soft tissue masses, most of which exhibit homogeneous density, and approximately 30% may contain focal calcifications (15, 16). However, central-type carcinoid tumors are characterized by nodules or masses arising from the trachea, lobar, or segmental bronchi, with affected distal bronchi often showing bronchiectasis or obstruction—features that help differentiate them from mediastinal ES. Central PSP typically presents as a round or oval soft tissue mass located near the pulmonary hilum or posterior mediastinum, with some lesions exhibiting gravel-like or patchy calcifications within (17). On contrast-enhanced CT, the mass appears as a map-like enhancement or progressive enhancement, and typical PSP may also show marginal vascular signs, halo signs, and tail signs, which help distinguish it from other tumors (18).

The diagnosis of ES requires histopathological examination. The proximal-type ES is characterized by large epithelioid cells arranged in a nodular pattern, frequently associated with central hemorrhage and necrosis within the nodules. Moreover, the tumor cells exhibit marked nuclear atypia, prominent nucleoli, and often focal or distinct rhabdoid morphology; occasional tumors show interstitial myxoid degeneration (2). ES is characterized by bidirectional mesenchymal and epithelial differentiation, so epithelial markers [such as CK and epithelial membrane antigen (EMA)] and mesenchymal markers (such as vimentin and CD34) are often positively expressed, while it is generally a negatively expressed for CD68, CD31, S100, HBM-45, INSM1, and Desmin (19). The histopathological examination of the current patient revealed a diffuse distribution of nodular epithelioid tumor cells under microscopic evaluation, accompanied by areas of necrosis and hemorrhage. Immunohistochemical analysis demonstrated positive expression of CK, vimentin, and CD34 in tumor cells, with negative immunoreactivity for INSM1, S100, and CD68—findings consistent with the established pathological diagnostic criteria for ES.

The management of ES primarily involves local radical resection of the tumor and regional lymph node dissection. Postoperatively, adjuvant radiotherapy and/or chemotherapy may be administered based on individual risk factors; however, their efficacy remains suboptimal (20). Compared with CES, PES is more prone to recurrence, early metastasis, and higher tumor-related mortality, resulting in a worse prognosis (19, 21). Due to the rarity of this disease, there are relatively few published studies on ES occurring in the mediastinum. Previous literature has reported a male child over 1 year of age with ES in the mediastinum who achieved remission following chemotherapy combined with radiotherapy, with no tumor recurrence observed during the 44-month follow-up period (22). Another study included a middle-aged female patient with mediastinal Ewing sarcoma (ES), focusing primarily on cytological analysis; however, no information was provided regarding the patient's treatment or prognosis (23). The overall chemotherapy response rate ranges from 20% to 33.3%. Commonly used regimens include epirubicin combined with ifosfamide, or gemcitabine combined with docetaxel (24, 25). The patient we reported had a recurrence of the tumor 4 months after surgery, revealing the highly malignant nature of the tumor.

## Conclusion

ES occurring in the mediastinum is rare, while our case suggests that it should be considered as one of the imaging differential diagnoses for mediastinal or near hilar tumors. It is prone to early recurrence after surgical resection, and the prognosis is poor. Therefore, understanding the clinical and imaging features of this rare disease is helpful for early diagnosis of the disease, and then early intervention to improve the prognosis.

## Data Availability

The original contributions presented in the study are included in the article/supplementary material, further inquiries can be directed to the corresponding authors.
